# Discourse-voice regulatory strategies in the psychotherapeutic interaction: a state-space dynamics analysis

**DOI:** 10.3389/fpsyg.2015.00378

**Published:** 2015-04-16

**Authors:** Alemka Tomicic, Claudio Martínez, J. Carola Pérez, Tom Hollenstein, Salvador Angulo, Adam Gerstmann, Isabelle Barroux, Mariane Krause

**Affiliations:** ^1^Faculty of Psychology, Universidad Diego PortalesSantiago, Chile; ^2^Faculty of Psychology, Universidad del DesarrolloSantiago, Chile; ^3^Psychology, Queen’s UniversityKingston, ON, Canada; ^4^Psychology, Pontificia Universidad Católica de ChileSantiago, Chile

**Keywords:** psychotherapeutic interaction, discursive positions, vocal quality patterns (VQP), state space grid (SSP), dynamic systems

## Abstract

This study seeks to provide evidence of the dynamics associated with the configurations of discourse-voice regulatory strategies in patient–therapist interactions in relevant episodes within psychotherapeutic sessions. Its central assumption is that discourses manifest themselves differently in terms of their prosodic characteristics according to their regulatory functions in a system of interactions. The association between discourse and vocal quality in patients and therapists was analyzed in a sample of 153 relevant episodes taken from 164 sessions of five psychotherapies using the state space grid (SSG) method, a graphical tool based on the dynamic systems theory (DST). The results showed eight recurrent and stable discourse-voice regulatory strategies of the patients and three of the therapists. Also, four specific groups of these discourse-voice strategies were identified. The latter were interpreted as regulatory configurations, that is to say, as emergent self-organized groups of discourse-voice regulatory strategies constituting specific interactional systems. Both regulatory strategies and their configurations differed between two types of relevant episodes: Change Episodes and Rupture Episodes. As a whole, these results support the assumption that speaking and listening, as dimensions of the interaction that takes place during therapeutic conversation, occur at different levels. The study not only shows that these dimensions are dependent on each other, but also that they function as a complex and dynamic whole in therapeutic dialog, generating relational offers which allow the patient and the therapist to regulate each other and shape the psychotherapeutic process that characterizes each type of relevant episode.

## Introduction

Thanks to research on developmental psychology, neuroscience, and attachment theory, a growing consensus has emerged highlighting the importance of affect regulation for the development of the self and of emotional interaction repertoires that determine relationships in life. These repertories are initially non-verbal but subsequently become systems of cognitions, emotions, and bodily responses which characterize ways of being with others. Furthermore, these repertoires or relational patterns, through the acquisition of language and the experience of multiple relationships, are embodied within a discourse (see Derek, 2006). This embodied relational history of an individual is expressed conjointly through verbal and non-verbal regulatory behaviors. These behaviors consist of explicit and implicit interactive processes, which are permanent and occur moment to moment. These are meant to organize the emotional and psychological experience of people in their relationship with others. They involve a number of psychological processes (e.g., mentalization), as well as non-verbal and verbal communication strategies. Particularly, in the psychotherapeutic context, each participant of the dyad is believed to be affected at every moment both by his/her own verbal and non-verbal self-regulation behaviors and by those of his/her partner, in a contingent and circular process of mutual regulation (Tronick, 1989; Tronick and Cohn, 1989; Schore, 1996; Tronick et al., 1998; Beebe and Lachmann, 2002; Fonagy et al., 2002; Beebe et al., 2005; Beebe, 2006).

Within the psychotherapeutic scenario, verbal regulatory behaviors are studied using the framework of psychotherapy as a discursive genre (Salvatore et al., 2010; Salvatore and Tschacher, 2012; Martínez et al., 2014a). Therapeutic discourse can be characterized by certain positions or perspectives adopted by speakers in the discourse as a whole that are equivalent to “positions of the self.” They are points of view expressed in an utterance, and it has been established that an utterance may contain more than one point of view, valuation, or position (Bakhtin, 1984). For example, we have described a positioning model for the patient’s and the therapist’s discursive positions. In the case of the patients, the *Reflexive*, *Dependent*, and *Independent* positions interact with each other in an internal dialog, and also in a dialog with others. We have observed the same in the therapists, who deploy three discursive positions: the* Proposer*, the* Professor*, and *Avoidant* (Martínez et al., 2014b).

On the other hand, it has been advanced that discursive positions are embodied within individuals in different manners (e.g., sound profiles, facial expression patterns, etc.), and are enacted within an interactive network in the psychotherapeutic dialog (Salgado et al., 2013). In other words, these positions are thought to be self-states which are structured in the language of an individual and which are expressed verbally and non-verbally. For example, it is believed that within the psychotherapeutic dialog the relationship between these discursive positions and their voice qualities constitutes an expression of regulatory and self-regulatory strategies of the participants (e.g., Osatuke et al., 2004; Tomicic et al., 2014). Discursive positions are thought to be expressed verbally using more than one vocal quality [e.g., Vocal Quality Patterns (VQP); Tomicic et al., 2011, 2014], which may be related to the idea that the implicit/primary level of experience (e.g., acoustic expressions) gives rise to a more integrative and explicit reflective-verbal level (e.g., Boston Change Process Study Group [BCPSG], 2002).

This study seeks to provide evidence of the emergence of configurations of recurring and stable discourse-voice regulatory strategies^1^ in patient–therapist exchanges in relevant episodes within each psychotherapeutic session as well as throughout the psychotherapy. Its central assumption is that discursive positions differ in terms of their prosodic characteristics in the therapeutic interaction according to their specific regulatory functions. In this regard, the hypothesis is that the patient and the therapist differently use each of their discourse-voice regulatory strategies according to their regulatory functions in different relevant episodes and moments over the psychotherapeutic process.

### The Triadic Model of Discursive Positioning

A multiplicity of discursive positions constitutes the identity of a person, not only in his/her dialog with another person, but also with the other positions of his or her own inner world (i.e., polyphonic metaphor; Bakhtin, 1986). Some of these positions could be under conscious control, temporally or permanently, and could dominate external and internal dialogs (Crits-Christoph et al., 1999; Gonçalves and Guilfoyle, 2006; Dimaggio and Stiles, 2007). Sometimes, this excessive control impedes dialog and the consideration of his or her other positions. Hence, excessive control could provoke rigidity in the way a person behaves and interacts with others in the world. Psychotherapy contributes to the modulation of and the dialog between the multiple positions of the patient. In this regard, the psychotherapeutic interaction helps activate the relationship between them, favoring those less conscious (or dissociated) to become more conscious and integrated for the patient. This is believed to allow a new discursive position to emerge: a metaposition with novel meanings (Stiles, 1999; Angus and McLeod, 2004; Bromberg, 2004; Hermans and Hermans-Jansen, 2004; Neimeyer and Buchanan-Arvay, 2004; Dimaggio and Stiles, 2007; Salvatore and Gennaro, 2012; Salvatore et al., 2012; Lehmann, 2013; Martínez and Tomicic, 2013).

We have described a triadic organization for the patient’s discursive positions (Martínez et al., 2014b). First, we identified a position called Reflexive, in which the patient is able to take a distant, but not disconnected, perspective of emotional situations, listening and critically looking at other positions while encouraging dialog between them in the manner of a metaposition (Bertau, 2008). Second, we described a position named Dependent, in which the patients subjectively position themselves as needy, weak, damaged, and/or vulnerable. Finally, we depicted a third position called Independent, which subjectively positions the patient as strong, self-sufficient, and/or as someone who does not need help from others (Martínez and Tomicic, 2013; Martínez et al., 2014b).

Similarly, we observed three discursive positions of the therapist. The first therapeutic discursive position was called the Proposer, in which the therapist subjectively positions him/herself as someone who shows what he/she observes, and offers the patient a new perspective, thus generating a dialogical space for the patient’s positions. In addition, therapists have a discursive position that we labeled the Professor, which is more dominant and monological, because it subjectively positions them as having a truth or knowledge that is imposed or taught to the patient as a sole alternative. Finally, we described a third position called Avoidant, in which the therapists subjectively distance themselves from the most problematic and difficult issues presented in the discourse of the patient, thereby closing any possibility of opening a dialogical space (Martínez et al., 2014b).

From an empirical perspective, the Positioning Model depicted seeks to establish regulatory strategies—verbal in this case—which are specific for psychotherapy. For example, the use of this model has shown that the patient adopts a Reflexive position as a metaposition that reveals other positions in him/herself (Dependent and Independent). Here, the Proposer position of the therapist has been shown to be very important, because it can reinforce this metaposition of the patient in a regulatory process that accomplishes a good therapeutic alliance and psychotherapeutic changes (Martínez et al., 2014b). However, these regulatory strategies are not only verbal; they occur moment by moment and at the same time in a non-verbal dimension that includes prosody.

### The Model of Vocal Quality Patterns

In the psychotherapeutic interaction, psychological meanings are exchanged not only through the participants’ speech, but also through the quality of their voices (Tomicic and Martínez, 2011; Tomicic et al., 2014). In this regard, the quality of the speaker’s voice may influence the emotional state of the listener. For instance, a voice that reflects the therapist’s relaxedness and confidence could calm the patient’s agitation and its associated emotions as reflected in the patient’s voice (Knoblauch, 2000, 2005). Similarly, it has been observed that, in the psychotherapeutic interaction, participants infer and cause emotions in each other through the prosody of their speech (Tomicic et al., 2009; Bauer et al., 2010).

To study the vocal patterns of self-regulation and mutual regulation between patient and therapist, we have developed a coding system called VQPs (Tomicic et al., 2011, 2014). VQPs are defined as a combination of specific vocal parameters—tone, intensity, duration, and pitch— in the utterances of speakers whose speech gives a specific impression to a listener, regardless of the contents transmitted. Six VQPs were identified and characterized: (a) *Report*, (b) *Connected*, (c) *Affirmative*, (d) *Reflexive*, (e) *Emotional-Expressive*, and (f) *Emotional-Restrained*. In addition, for utterances in which these VQP codes do not apply, the following categories were created: (g) *Full Pause*, (h) *Overlapping*, and (i) *Not Codable*. As shown in **Table 1**, each of the VQPs is described according to the manner in which it impresses the person who is listening.

**Table 1:** Characterization of vocal quality patterns.

VQP	Phenomenological characterization
Report	It adds to the speech the quality of *something already known*, of a speech disconnected from what is being said and/or of a certain emotional distance. It sounds as if the speaker was reporting, narrating, or exploring content without any emotional involvement. In this pattern, the central element is the listener’s impression of a disconnected speech. *Main Vocal Parameters*: INTENSITY: increased volume and large variations; DURATION: speed augmented.
Connected	It conveys the quality of being oriented toward the other (the partner in the dialog) and of being carefully prepared while it is uttered. In this pattern, the central element is the listener’s impression of an elaborative speech geared toward the partner in the dialog. *Main Vocal Parameters*: TONE: dynamic-agogic accent, half-suspended anti-cadence at the end of the phrase; INTENSITY: increased volume, sustained-crescendo dynamics, and small variations.
Affirmative	It conveys the quality of certainty and conviction. It sounds as if the speaker were teaching or instructing the listener, or as if he/she were very sure of what he/she is saying. In this pattern, the central element the listener’s impression of a secure and instructive speech. *Main Vocal Parameters*: TONE: dynamic-tonic accent and suspended end of phrase; INTENSITY: dynamics sustained-crescendo; DURATION: Hard vocal attack.
Reflection	It conveys the quality of being directed *toward oneself* (the speaker). It sounds as if the speaker was connected with her/his internal world or in a dialog with her/himself. In this pattern, the central element is the listener’s impression of an introverted speech. *Main Vocal Parameters*: TONE: dynamic-agogic accent and half-suspended cadence at the end of the phrase; INTENSITY: decreased volume and small variations; DURATION: speed reduced.
Emotional-expressive	It conveys affection and/or the sensation that the speech has a heavy emotional load. It sounds like the speaker’s emotion (joy, anger, sadness, fear, etc.). In this pattern, the central element is the listener’s impression of an emotionally charged speech, regardless of the type of emotion. *Main Vocal Parameters*: TIMBRE: Clear/Bright; Clear/Opaque; Dark/Bright; and Dark/Opaque.
Emotional-restrained	It conveys affection and/or the sensation that the speech has a heavy emotional load. However, even though in this case the speaker’s emotion is not audible, what does impress the listener is an effort to contain her/his *emotion*. In this pattern, the central element is the listener’s impression of suffocation and control to avoid being overwhelmed by emotion. *Main Vocal Parameters*: DURATION: speed decreased, non-fluid pace, and long pauses.
**Exclusion Categories for VQPs**			
Overlapping	It is an instance of simultaneous speech, which, in VQP coding, makes it impossible to distinguish the vocal characteristics of the participants in a full segment or speaking turn. When coding this conversation phenomenon, the overlapping of the actors is noted.
Full pause	Short utterances with para-verbal content (hmm, aha, okay). They are usually ways of agreeing, showing attention, disagreeing, or displaying the wish to end a conversation. Their meaning depends mainly on the context and on certain vocal characteristics of the utterance; however, due to their brevity, they are hard to analyze in terms of the vocal parameters that define the VQPs described.
Non codable	These are units of analysis which do not meet the phenomenological characteristics and the parameters of the VQPs. This label can also be applied to the cases in which the recording is not completely audible due to ambient noises, mispronunciations, or other errors by the speakers. They are neither full pauses nor instances of overlapping.

In a previous study (Tomicic et al., 2014), we were able to observe the process of change embodied in the expressive vocal styles of the participants of psychotherapeutic dyads, and to uncover regulatory sequences between them. This showed us that it is possible to detect the emergence of regulatory patterns in therapeutic interaction in the form of vocal expressions, and that these patterns are involved in the process of change in psychotherapy. Based on the assumption that these vocal qualities impress the patient and the therapist who are listening in the same way that they impress the coders, these results may imply that, in psychotherapeutic practice, the participants not only take into account the content of the speech they produce and listen to, but also unconsciously integrate prosody in their regulatory behaviors as another dimension of their experience of the psychotherapeutic encounter.

### The Micro-Process Analysis of the Relationship Between Discourse and Voice as Regulatory Strategies in the Psychotherapeutic Interaction

Sequential analyses have shown us that the association of two different discursive positions or the association of two different VQPs can be interpreted as micro-regulatory strategies (Martínez, 2011; Tomicic et al., 2014). We have identified two types of these regulatory strategies: self-regulatory strategies (a sequence of two different discursive positions or a sequence of two different VQPs that take place in the same patient or therapist utterance) and mutual regulation strategies (a sequence of two different discursive positions or a sequence of two different VQPs that correspond to the interaction between the members of the therapeutic dyad; Martínez and Tomicic, 2013; Tomicic et al., 2014). These analyses revealed different discursive and vocal micro-regulatory strategies depending on the type of relevant episode considered (i.e., Change Episodes and two types of non change episodes: Stuck Episodes or Rupture Episodes). For example, in a single case study with a long-term psychoanalytically oriented therapy, it was observed that the Reflexive position of the patient followed by the Proposer position in the therapist constituted a mutual regulatory strategy more prevalent in Change Episodes compared to Rupture Episodes (Martínez and Tomicic, 2013). In addition, another study showed that the Connected VQP of the patient followed by the same VQP of the therapist constituted a mutual regulatory strategy that was more prevalent in Change Episodes compared to Stuck Episodes (Tomicic et al., 2014). In the present study only Change Episodes and Rupture Episodes were analyzed.

Even though we have observed the deployment of micro-regulatory strategies, the scope of these observations cannot account for the dynamics involved in the emergence and self-organization of configurations of discursive or vocal regulatory strategies of the psychotherapeutic process. That is to say, our previous analyses were not pertinent enough to approach the study of patient–therapist regulation in terms of discourse and prosody as aspects of a dynamic system, considering it in the therapeutic context as a set of co-occurring elements that have clinical value (Salvatore and Tschacher, 2012; Hollenstein, 2013). In this regard, the purpose of the current study was to explore the dynamics associated with the emergence of configurations of recurring and stable regulatory strategies in patient–therapist interaction in terms of discourse and voice associations over time (Osatuke et al., 2004), in two different relevant episodes (Change Episodes and Rupture Episodes), and within psychotherapeutic sessions. Following Fogel (2006, 2011), our intention was to seek individual recurrent and stable discourse-voice associations (i.e., microscopic level) that lead to the emergence of patterns involving these associations (i.e., macroscopic level). Specifically, our aims were:

– To observe recurrent and stable discourse-voice associations that could be interpreted as regulatory strategies of patient–therapist interaction.
– To determine differences in the use of these discourse-voice regulatory strategies in Change Episodes and Rupture Episodes, and session to session.
– To identify specific groups of recurrent and stable discourse-voice regulatory strategies that could be interpreted as regulatory configurations.
– To determine differences in the prevalence of these regulatory configurations in Change Episodes and Rupture Episodes, and session to session.

We used the dynamic systems theory (DST) approach (Kaplan and Glass, 1995; Fogel, 2011), specifically the concept of *attractor*. From this perspective, the behavior of a system can be understood as a path within a landscape with its topology, in which the system gets stabilized in some states of the set of possible states of that territory. Therefore, attractors are recurring and stable states where systems remain more often and to which they tend to return (Salvatore and Tschacher, 2012).

In this case, the association between discourse and voice was analyzed with the state space grid (SSG) method, a graphical tool based on a dynamic system approach (Hollenstein, 2013). Phenomena that involve two synchronous variables are plotted in a two-dimensional space as a trajectory or sequence of states that move from cell to cell on the grid. SSGs can be used to identify which states are more frequent and stable (i.e., attractors). In our research, the system comprises the therapeutic activity in the relevant episodes considered: Change Episodes (Krause et al., 2006) and Rupture Episodes (Safran and Muran, 1996). In addition, the possible states through which this system moves reflect the combinations of the discursive positions of patients and therapists with each VQPs. Each of the system’s trajectories accounts for a different state sequence as part of the psychotherapeutic process.

Considering previous studies (see above), and the conceptual association between some discursive positions and certain VQPs (e.g., the Reflexive position with the Connected VQP; see above), it was expected that the identified attractors would empirically reveal the presence of such relationships. Thus:

(1) It was expected that, for patients, there would be three discourse-voice regulatory strategies working as attractors: Reflexive position with Connected VQP; Dependent position with Emotional VQP (Expressive and Restrained conjointly), and Independent position with Affirmative VQP. For therapists, two discourse-voice regulatory strategies working as attractor were expected: Proposer position with Connected VQP and Professor position with Affirmative VQP.
(2) The regulatory strategies working as attractors Reflexive position with Connected VQP (patient’s) and Proposer position with Connected VQP (therapist’s) were expected to be more frequent in Change Episodes.
(3) Different configurations of discourse-voice regulatory strategies working as attractors were expected to emerge in the patient and the therapist, each having different values for therapeutic activity.
(4) These different configurations of discourse-voice regulatory strategies working as attractors were expected to be present in dissimilar proportions in Change Episodes and Rupture Episodes.

## Materials and Methods

### Participants

Change Episodes (*N* = 67) and Rupture Episodes (*N* = 86) were considered as interactional scenarios, and were taken from five therapies. The mean age of patients was 34.7 years (SD = 12.1), and 80% were female. Patients received an average of 32.8 (SD = 6.4) therapy sessions, with a psychodynamic or cognitive focus, in a context of outpatient treatment. The therapists, three males and two females, had between 3 and 15 years of professional experience. All the treatments were evaluated by means of an outcome measurement using the Outcome Questionnaire 45.2 (OQ-45.2, Lambert and Burlingame, 1996; von Bergen and de la Parra, 2002; see **Table 2**). Successful therapies were defined as those that met the criterion of resulting in a reliable change index (RCI) of 15 or more (von Bergen and de la Parra, 2002). According to this instrument, three of five therapies were successful.

**Table 2:** Description of the psychotherapeutic processes and relevant episodes.

	Patient	Therapist	Diagnosis	Modality	Initial OQ	RCI	Session	*N*	Change E. *fc* (%)	Rupture E. *fc* (%)
1	Female	Female	Adaptive disorder	Psychodynamic	80	6	88^a^	45^a^	19 (42.2%)	26 (57.8%)
2	Male	Male	Anxiety disorder	Cognitive-behavioral	50	28	11	16	6 (37.5%)	10 (62.5%)
3	Female	Male	Depression	Psychodynamic	49	7^b^	31^b^	37	23 (62.2%)	14 (37.8%)
4	Female	Male	Personality disorder	Cognitive-behavioral	55	15	15	45	11 (24.4%)	34 (75.6%)
5	Female	Female	Adaptive disorder	Psychodynamic	75	19	19	10	8 (80.0%)	2 (20.0%)

The diagnosis was reported by each therapist. *E* = Episodes. Outcome: Successful therapies were defined as those that met the criterion of attaining a reliable change index (RCI = 15 or more; von Bergen and de la Parra, 2002). ^a^The first 20 sessions of the full psychotherapeutic process were analyzed in this study; 45 Episodes were identified in this period of the psychotherapeutic process. ^b^This psychotherapy was restarted after 4.5 months of suspension; the 31 sessions that were coded correspond to the first period of therapy (RCI was measured at session 31).

### Procedures

All sessions were video and audio recorded. Patients and therapists were extensively informed before commencing therapy, and all of them consented to video and audio recordings and to data collection at all times. Also, all participants provided a written informed consent concerning the use of their data for research purposes. The study was approved by the research ethics committee of Universidad Diego Portales (CEI-UDP, Chile).

The sessions were coded using two sequential procedures: first, to identify relevant episodes; second, to analyze the discourse and vocal behavior of the participants in said episodes.

#### Determination of Relevant Episodes

The units of analysis used were relevant episodes. These are special segments of the therapeutic session that are chosen from a theoretical point of view. These episodes make it possible to understand the connection between the therapeutic exchange and its outcome (Elliott, 1984; Timulak, 2007). In this study, Change Episodes (Krause et al., 2006, 2007) and Rupture Episodes (Safran and Muran, 1996, 2000, 2006) were used.

The method for determining Change Episodes is based on the subjective notion of generic change (Krause, 2005; Krause et al., 2007). Subjective change is operationalized by means of “Generic Change Indicators” (Krause et al., 2006), which make it possible to identify a change moment based on its content (see Krause et al., 2007). For its part, a Change Episode is an interaction segment where a change moment takes place. In the rating procedure, this moment marks the end of the episode. At this point, a rater establishes the beginning of the episode by tracking back when the participants start conversing about the content of the change (Krause, 2005).

For the identification of Rupture Episodes, we used the Rupture Resolution Rating System Manual (Eubanks-Carter et al., unpublished), which specifies communication markers derived from the two main types of rupture of the alliance indicated by Safran and Muran (1996, 2000, 2006): withdrawal and confrontation. With respect to the temporal delimitation of Rupture Episodes, their beginning was established by the very first communicational hints of rupture, while their end was established by the very first hints of their resolution or overcoming (Martínez, 2011).

#### Coding of Discursive Positions

This analysis consists in identifying the positions that appear in the discourse of each participant and which shed light on his/her way of being, interacting with others, and interpreting the world. These positions are identified in the transcripts of the episodes by paying attention to the patient’s and the therapist’s discourse and by depicting the main discursive features present in the speech of both participants.

The identification of discursive positions was carried out in a previous study (Martínez et al., 2014b,c) using a device that considered two analytical steps:

##### Step 1: identification and characterization of discursive voices

The first three sessions of the five therapies were coded. Each speaking turn was read and coded with the aim of answering the question “What are the participants talking about?” This made it possible to identify recurrent enunciators in the speech of the patient and the therapist in each therapy. These enunciators were preliminarily labeled according to their main predicate, which resulted in a set or repertoire of specific discursive voices for each therapy. Discursive markers were identified and a phenomenological description of each of the discursive voices was performed. The purpose was to answer the question “How do the discursive voices speak?” The discursive markers considered were (a) subject of the utterance, (b) subject of the enunciation, and (c) modalizers (see Martínez et al., 2014a).

##### Step 2: categorization of the set of discursive voices and labeling of each category as a discursive position

The discursive voices of each actor were grouped into inclusive categories of a higher abstraction level. Each category was labeled according to the subjectivity involved in that specific repertoire of discursive voices. The purpose was to answer the question “From which perspective does each voice speak?” For instance, in Therapy 1, the repertoire of discursive voices of the patient constituted by “hopeless,” “distrustful,” “pampered,” “rejectable,” and “abused” were interpreted as the position “Dependent.” This choice was made because, in this set of voices expressed in her speech, the patient subjectively takes the place of a defenseless little girl, someone who has been abused and harmed, and who is rejectable and unable to make decisions or think for herself.

The discursive positions determined through this process were used to code the relevant episodes of each of the five therapies. The transcriptions of each of the relevant episodes were coded by two raters using ATLAS.ti 7.0.5 (1993-2015), a type of Computer Assisted Qualitative Data Analysis Software (CAQDAS).

#### Coding Vocal Quality Patterns

Each episode was analyzed by raters trained in the use of the VQP coding system (Tomicic et al., 2011). With the VQP coding system, the raters categorized the patient’s and the therapist’s speech in terms of vocal quality. This system identifies six mutually exclusive VQPs: (a) Report, (b) Connected, (c) Affirmative, (d) Reflexive, (e) Emotional-Expressive, and (f) Emotional-Restrained. Also, for the utterances in which the VQP coding does not apply, the following categories were created: (g) Full Pause, (h) Overlapping, and (i) Non Codable (see **Table 1**).

The VQP coding procedure was carried out in four analytic steps for each episode: (1) Listening to the full episode, so as to become familiar with the timbre of the participants’ voices; (2) Listening from the start of the episode, reading the text speaking turn by speaking turn, and performing a preliminary segmentation considering changes or breakdowns in vocal quality as revealed by changes in a vocal parameter; (3) Listening from the start of the selected episode, speaking turn by speaking turn and segment by segment, and performing a preliminary coding considering the phenomenological description of the VQPs; and (4) Listening from the start of the selected episode to confirm or discard the presence of the VQP coded in step three considering the auditory perception of the vocal quality parameters involved.

#### Coding Validation Process

##### Relevant Episodes Coding

For the selection and temporal delimitation of the Change Episodes and Rupture Episodes, five pairs of coders trained by the Chilean Research Program on Psychotherapy and Change analyzed videotapes and transcriptions of the therapeutic sessions and carried out an intersubjective validation procedure. This procedure is a process in which the observations by a researcher or rater are compared with the independent observations of other researchers or raters. The validation of observations is attained through consensus or agreement between these different perspectives (see Flick, 2009). In this case, inter-rater reliability was not calculated because it was considered that the independent coding of the episodes was only carried out in preparation for their intersubjectively validated coding.

##### Discursive Positions and VQP Coding

In order to ensure the quality of the data resulting from these two coding processes, a couple of raters trained in the use of each of the systems coded all the relevant episodes independently; afterward, their codings were combined to generate a single consensus coding through an intersubjective validation procedure.

In addition, as a checking procedure, a reliability study was performed for the discursive positions and VQPs, using Cohen’s Kappa (Cohen, 1968) to measure the independent raters’ agreement. We considered all of the episodes (*n* = 153). Discursive positions coding (6575 segments) resulted in *k* = 0.762, *p* < 0.05. On the other hand, VQP coding (4553 segments) resulted in *k* = 0.658, *p* < 0.05.

#### Identification of Regulatory Strategies (by Means of SSGs)

To account for the discourse-voice association, the independent data of discourse and VQPs obtained were matched at the level of turn-taking. If an instance of turn-taking occurred across more than one segment, the correspondence of the categories of discourse and voice was determined by the researchers using the transcription of the episode, creating new segments if necessary in order to adequately make the two variables coincide.

Once the joint database was constructed, it was imported into the GridWare SSG software (Lamey et al., 2004). The SSG allowed the joint analysis of the data, considering both the discursive and the prosodic behavior of the patient or the therapist during the course of the episodes. As shown in **Figure 2**, the X-axis represents the categories of the discursive positions of the patient and the therapist, while the Y-axis represents the categories of the VQPs for both. In each cell of the SSG, the size of the plot point represents the *number of visits* [Rate of Visit (RV)] of a given association of a Discursive Position with a VQP of the patient or the therapist, which makes it possible to identify the attractors.

### Data Analyses

With the purpose of determining the attractors, that is to say, the most recurring and stable discourse-voice regulatory strategies, the data obtained with the SSG were analyzed using the *Winnowing* technique (Lewis et al., 1999; Hollenstein, 2013). Afterward, in order to determine the configurations of discourse-voice regulatory strategies working as attractors, a cluster analysis was performed using SSPS-17 (SPSS Inc., 2008). Finally, to compare the prevalence of the discourse-voice regulatory strategies and their configurations in Change Episodes and Rupture Episodes, Logistic Hierarchical Regression analyses were conducted using HLM 7.0 (Raudenbush et al., 2011).

## Results

### Discourse-Voice Regulatory Strategies: The Attractors

The RVs within each episode was used to identify Discourse-Voice Regulatory Strategies that were defined as attractors. Following a conceptual model named “*Virginia Model,*” which establishes the association of verbal and non-verbal behaviors in a nested manner (Martínez et al., 2014b), attractors were determined with respect to each discursive position^2^ (i.e., the X-axis of the SSG; see **Figure 2**). In this conceptual model, the discursive position is the explicit dimension of regulation with the other, which makes it possible to understand the meaning of the implicit and non-verbal dimensions of the interaction. Thus, the model considers two levels of analysis. The first one concerns the analysis of the non-verbal profiles of the discursive positions of each member of the therapeutic dyad. The second involves the microanalysis of the regulatory function of the combined manifestations of the discursive and non-verbal expressions of patient–therapist interaction (i.e., discourse-voice regulatory strategies) within Change Episodes and Rupture Episodes of the psychotherapeutic process (Martínez et al., 2014b).

In order to identify the attractors (i.e., recurrent and stable discourse-voice regulatory strategies), the *Winnowing* technique (Lewis et al., 1999; Hollenstein, 2013) was used. This method consists of a series of runs, starting with all occupied cells and shifting to a smaller set of cells each time. A mean-squared *heterogeneity* value for the whole set of cells, corresponding to each Discursive Position combined with the seven VQPs (1 × 7 grids), was calculated with the following formula:

$$Heterogeneity_{j}=\frac{\Sigma {\left(Observed_{i}-Expected_{j}\right)}^{2}/Expected_{j}}{\#\,\;of\,\;\;Cells_{j}}$$

**FIGURE 1:**
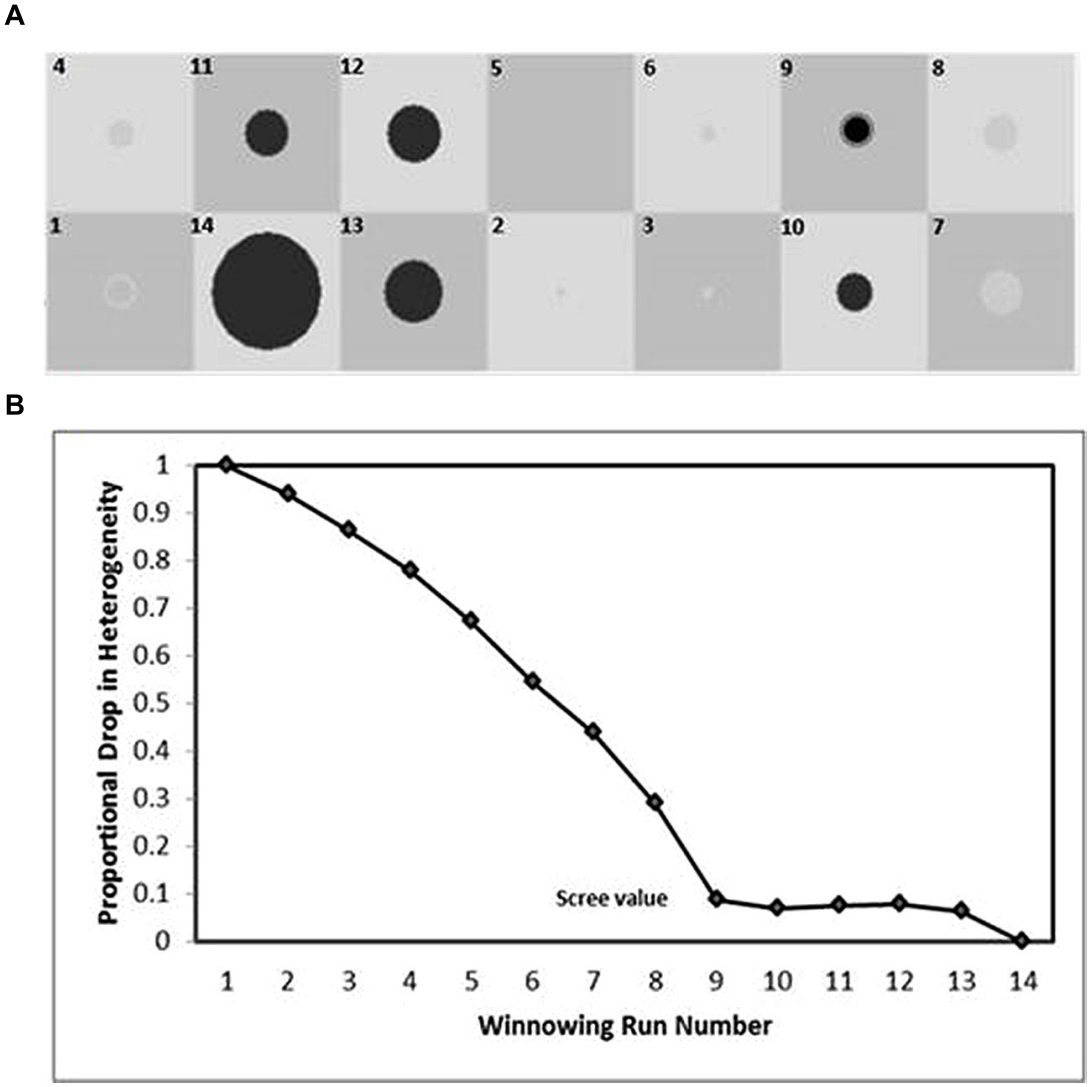
**Identifying attractors by using the Winnowing procedure (based on Lewis et al., 1999).** Each winnowing run denotes grid cells with greater visits whose removal decreases the heterogeneity of the set **(B)**. After the steepest drop in heterogeneity (scree value), the remaining cell or cells (in black) comprise the attractors **(A)**.

Then the cell with lowest visits value was excluded, and the calculation was repeated on the next subgroup of cells. This procedure was repeated eliminating one cell visits value at a time, until only the attractor cells remained. As is exemplified in a hypothetical 2 × 7 grid in **Figure 1**, the mean square for heterogeneity dropped from run to run as the subgroup of cells got smaller (see **Figure 1B**). By means of visual inspection the attractors were identified as the most homogeneous group of cells, shown in this case by the flattening of the scree plot at run nine (see **Figure 1B**). Eliminating any of the cells that remained at run nine would not decrease heterogeneity and hence, in this example, six cells are considered as attractors (see **Figure 1A**).

In the case of the patients, the cells Reflexive position-Connected VQP and Reflexive position-Affirmative VQP were visited more frequently than the other combinations (see **Table 3**). Therefore, as was expected (see hypothesis 1), the combination Reflexive position-Connected VQP is a discourse-voice regulatory strategy working as attractor; for its part, the combination Reflexive position-Affirmative VQP was an unexpectedly discovered attractor.

**Table 3:** Total visits for each discursive position-VQP combination in patients and therapists.

Discursive positions/VQPs	Report	Connected	Affirmative	Reflexive	Emotional	Full pause	Overlapping	Total
**Patient**
Reflexive	46 (9.97%)	184^∗^ (39.91%)	87^∗^ (18.87%)	27 (5.85%)	50 (10.84%)	47 (10.19%)	20 (4.33%)	461
Dependent	93^∗^ (22.51%)	93^∗^ (22.51%)	51 (12.34%)	18 (4.35%)	120^∗^ (29.05%)	25 (6.05%)	13 (3.14%)	413
Independent	50 (10.46%)	131 (27.40%)	103 (21.54%)	15 (3.13%)	80 (16.73%)	55 (11.50%)	44 (9.20%)	478
**Therapist**
Proposer	53 (7.48%)	307^∗^ (43.36%)	146 (20.62%)	12 (1.69%)	10 (1.41%)	132 (18.64%)	48 (6.77%)	708
Professor	46 (10.43%)	154^∗^ (34.92%)	118^∗^ (26.75%)	7 (1.58%)	15 (3.40%)	57 (12.92%)	29 (6.57%)	441

The asterisks indicate the identified attractors. *N* = 153 episodes.

In the case of the Dependent discursive position, the strategies Dependent position-Report VQP, Dependent position-Connected VQP, and Dependent position-Emotional VQP received more visits than the other discourse-voice regulatory strategies. Thus, as was expected (see hypothesis 1), the combination Dependent position-Emotional VQP was found to be an attractor, while the other two combinations were unexpectedly discovered attractors (see **Table 3**).

Finally, for the Independent discursive position, no attractors were identified, because neither of the heterogeneity values of the cells showed a significant drop or ‘scree’ (see **Table 3**). Therefore, in this case the hypothesized attractor was not confirmed (see hypothesis 1).

**FIGURE 2:**
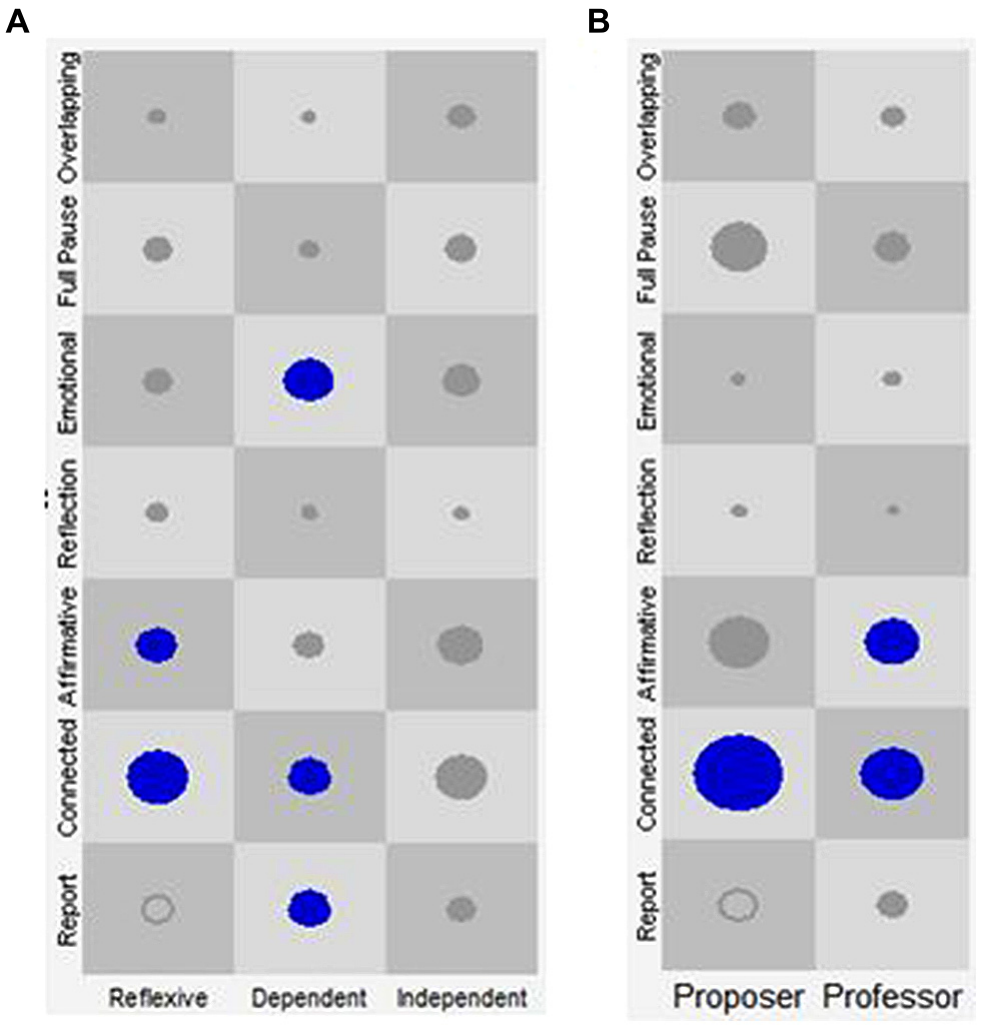
**Discourse-voice regulatory strategies of patients and therapists regardless of episode type.** The *X*-axis represents the discursive positions of the patient (**A**: Reflexive, Dependent, and Independent) and of the therapist (**B**: Proposer, and Professor). The *Y*-axis represents the Vocal Quality Patterns (VQP) for patient and therapist. Each one of the cells represents all the state-spaces resulting from the combination of the discursive positions with the VQPs. The circles and their size represent the visits in each cell and their frequency, respectively. Only the circles in blue represent the identified attractors.

In sum, the attractors identified for the patients were: (a) Reflexive position-Connected VQP, (b) Reflexive position-Affirmative VQP, (c) Dependent position-Report VQP, (d) Dependent position-Connected VQP, and (e) Dependent position-Emotional VQP (see **Figure 2A**).

In the case of the therapists, the results presented in **Figure 2B** and **Table 3** indicate that the Proposer position-Connected VQP regulatory strategy received more visits than the other combinations. Therefore, as was expected (see hypothesis 1), this combination was an attractor. For the Professor discursive position, the combinations Professor position-Connected VQP and Professor position-Affirmative VQP received more visits than the other discourse-voice strategies. Thus, as was expected (see hypothesis 1), the combination Professor position-Affirmative VQP was found to be an attractor. On the other hand, the combination Professor position-Connected VQP was an unexpected attractor (see **Table 3**).

In brief, the attractors identified for the therapists were (a) the combination Proposer position-Connected VQP and (b) the combinations Professor position-Connected VQP or Affirmative VQP (see **Figure 2**).

### Discourse-Voice Regulatory Strategies: Attractors in Relevant Episodes

The discourse-voice regulatory strategies working as attractors identified were compared according to their presence in Change Episodes and Rupture Episodes. Thus, the dependent variable was the presence (0 = absence or 0 RV; 1 = presence or 1 RV or more) of each of the attractors at the episode level (the discourse-voice strategies marked with an asterisk in **Table 3**). The probability of each of the attractors was compared according to the type of episode considered. A Logistic Hierarchical Regression analysis (using HLM version 7.0, Full-PQL estimation method, Bernoulli distribution at Level-1) in a 2-Level model was used for establishing the differences between the attractors by type of episode.

In the model, the episodes (Level-1) were nested in the patient (Level-2)^3^. The type of Episode was the predictor at Level-1 (0 = Rupture Episode and 1 = Change Episode). The Level-2 predictors were Initial Patient Functionality^4^ (Functional, indicating the level of functioning of the patient when starting the therapy; 0 = Beginning the psychotherapy in the dysfunctional population and 1 = Beginning the psychotherapy in the functional population), and Reliable Change Index in the Patient (RCI, indicating the outcome of the therapy; 0 = without RCI and 1 = with RCI)^5^.

Separate HLM analyses were conducted for each selected discourse-voice strategy at the episode level (eight attractors). Three steps were followed for each analysis:

(a) A fully unconditional model was fitted in order to estimate dependent variable reliability and the adequacy of the multilevel analysis.
(b) The type of Episode was included in the Level-1 equation and modeled as a random effect in order to determine whether the coefficients varied among patients. If there was no variability to explain, its variance was fixed at zero.
(c) Finally, Initial Patient Functionality and/or Reliable Change Index in the Patient were included as predictors at the Level-2 intercept and/or slope (Type of Episode). Whenever these predictors did not explain significant variances of the Level-2 equations, they were also dropped out of the model. **Tables 4** and **5** present the final models of each discourse-voice regulatory strategy at the episode level.

**Table 4:** Discourse -voice regulatory strategies of patients according to type of episode (HLM).

**Fixed Effects^**a**^**	**Reflexive-connected^**c**^**	**Reflexive-affirmative^**d**^**	**Dependent-report^**d**^**	**Dependent-connected^**c**^**	**Dependent-Emotional^**e**^**
	**Coefficient (SE)** **Odds (95% CI)**	**Coefficient (SE)** **Odds (95% CI)**	**Coefficient (SE)** **Odds (95% CI)**	**Coefficient (SE)** **Odds (95% CI)**	**Coefficient (SE)** **Odds (95% CI)**
Intercept (γ_00_)	-1.04 (0.53) 0.35 (0.08–1.54)	-1.58 (0.31)^∗^ 0.21 (0.08–0.56)	-1.69 (0.56) 0.18 (0.03–1.09)	-1.67 (0.57) 0.51 (0.10–2.45)	-1.15 (0.27)^∗^ 0.32 (0.13–0.75)
Initial patient functioning (γ_01_)^b^	–	2.93 (18.81)^∗^ 18.81 (1.65–215.01)	-3.63 (1.10)^∗^ 0.27 (0.001–0.89)	–	-1.60 (0.42)^∗^ 0.20 (0.05–0.77)
Type of episode (γ_10_)	2.09 (0.43)^∗∗∗^ 8.09 (3.45–19.01)	1.44 (0.40)^∗∗∗^ 4.21 (1.90–9.32)	-0.44 (0.50) 0.65 (0.24–1.73)	-0.32 (0.40) 0.73 (0.33–1.60)	0.37 (0.52) 1.32 (0.31–5.65)
	
**Random variance components**				
Level-2 Intercept(*u*_0_)	0.939^∗∗∗^ 31.09 (4)	0.001 4.43 (3)	0.737 14.03 (3)	1.220 26.21 (4)	0.012 4.78 (3)
Level-2 type of episode(*u*_1_)	–	–	–	–	0.53^∗^ 10.30 (43)

Level-1 *N*= 153, Level-2 *N*= 5. Type of episode, 0 = Rupture Episodes and 1 = Change Episodes. Functional, 1 = Beginning the psychotherapy in the functional population and 0 = Beginning in the dysfunctional population. ^a^Gamma (γ) coefficients in fixed effects and variance components in random effects. Standard errors (SE) follow parameter estimates in parentheses and 95% Confidence Interval (CI) Odds Ratio follow Odds Ratio estimator in parentheses (Fixed Effects). Ch^2^ and *df* below variances in parentheses (Random effects). ^b^Initial Patient Functioning centered around the grand mean. Final Models: ^c^Log (Probability of Dependent Variable) = γ_00_ + γ_10_^∗^Type of Episode+ u_0_; ^d^Log (Probability of Dependent Variable) = γ_00_ + γ_01_^∗^Functional + γ_10_^∗^ Type of Episode+ u_0_; ^e^Log (Probability of Dependent Variable) = γ_00_ + γ_01_^∗^Functional + γ_10_^∗^Type of Episode+ u_0_+ u_1_. ^∗^*p*< 0.05, ^∗∗∗^*p*< 0.001.

**Table 5:** Discourse-voice regulatory strategies of therapists according to type of episode (HLM).

	Discursive position-VQP (Therapists)		
Fixed effects^a^	Proposer-connected^c^	Professor-affirmative^d^	Professor–connected^e^
	Coefficient (SE) odds (95% CI)	Coefficient (SE) odds (95% CI)	Coefficient (SE) odds (95% CI)
Intercept (*γ*_00_)	0.86 (0.56) 2.36 (0.49–11.35)	-0.09 (0.27) 0.90 (0.38–2.17)	0.47 (0.27) 1.60 (0.75–3.42)
Initial patient functioning (*γ*_01_)^b^	–	2.06 (0.49)^∗^ 7.78 (1.62–38.27)	–
Type of Episode (*γ*_10_)	1.20 (0.45)^∗^ 3.33 (1.34–8.27)	-0.07 (0.73) 0.93 (0.13–7.02)	-0.51 (0.63) 0.60 (0.14–3.47)
**Random variance components**
Level-2 Intercept(*u*_0_)	1.12^∗∗∗^ 23.99 (4)	0.09 3.13 (3)	0.11 5.31 (4)
Level-2 Type of Episode(*u*_1_)	–	1.79^∗∗^ 16.03 (4)	1.32^∗∗^ 14.23 (4)

Level-1 *N*= 153, Level-2 *N*= 5. Type of episode, 0 = Rupture Episodes and 1 = Change Episodes. Functional, 1 = Beginning the psychotherapy in functional population and 0 = Beginning in the dysfunctional population. ^a^ Gamma (γ) coefficients in fixed effects and variance components in random effects. Standard errors (SE) follow parameter estimates in parentheses and 95% Confidence Interval (CI) Odds Ratio follow Odds Ratio estimator in parentheses (Fixed Effects). Ch^2^ and *df* below variances in parentheses (Random effects). ^b^Initial Patient Functioning centered around the grand mean. Final Models: ^c^Log (Probability of Dependent Variable) = γ_00_ + γ_10_^∗^Type of Episode+ u_0_; Log (Probability of Dependent Variable) = γ_00_ + γ_01_^∗^Functional + γ_10_^∗^Type of Episode + u_0_+ u_1_; ^e^Log (Probability of Dependent Variable) = γ_00_ + γ_10_^∗^Type of Episode+ u_0_ + u_1_.

^∗^*p* < 0.05, ^∗∗^*p* < 0.01, ^∗∗∗^*p* < 0.001

The results indicated that the regulatory strategy working as attractor Reflexive position-Connected VQP was more likely to be used in Change Episodes than in Rupture Episodes (Odds ratio 8.09, 95% CI 3.24; 19.01), thereby confirming hypothesis 2. The same was observed for the attractor Reflexive position-Affirmative VQP (Odds ratio 4.21, 95% CI 1.90; 9.32).

Finally, a comparison of the therapists’ use of discourse-voice regulatory strategies working as attractors at the episode level revealed that the Proposer position-Connected VPQ regulatory strategy was the only one whose presence was significantly different in Change Episodes and Rupture Episodes (see **Table 5**). Therefore, as was expected (see hypothesis 2), therapists were more likely to use the attractor Proposer position-Connected VQP in Change Episodes than in Rupture Episodes (Odds ratio 3.33, 95% CI 1.34; 8.27).

In these models, Initial Patient Functioning was included as a control variable. Nevertheless, the results indicate that the Dependent position-Report VQP and the Dependent position- Emotional VQP attractors were more likely to be used as regulatory strategies by the patients who began the psychotherapy in the dysfunctional population. The opposite was true for the attractors Reflexive position-Affirmative VQP when used by the patient and Professor position-Affirmative VQP when used by the therapist. These strategies were applied more frequently when the patients began therapy in the functional population.

### Configurations of Discourse-Voice Regulatory Strategies: Identification of Patterns of Attractors

To test hypothesis 3, a Cluster analysis was performed to determine emerging patterns of attractors (see Fogel, 2006, 2011), that is to say, patterns of recurrent and stable discourse-voice regulatory strategies within relevant episodes. Thus, this analysis was performed considering the total number of episodes as the subject to be classified (*N* = 151^6^). Using the classification command K-Means (Quick Cluster in SPSS-17.0) 3-, 4-, and 5-cluster solutions were explored. The 4-cluster solution was selected using as criterion the parsimony and interpretability of each cluster.

Each cluster was qualitatively interpreted according to its global regulatory configuration, especially the specific attractors of each one. The *Winnowing* technique (explained above) was used to identify the recurrent and stable discourse-voice regulatory strategies for each regulatory configuration. On this occasion, the mean-squared *heterogeneity* value was calculated for the whole set of cells corresponding to the discursive positions of the patient combined with the seven VQPs (3 × 7 grids) and for the set of cells corresponding to the discursive positions of the therapist also combined with the VQPs (2 × 7 grids). Therefore, each of these configurations represents a group of discourse-voice strategies working as attractors that shape an interaction as a specific form of mutual regulation between patient and therapist.

#### Description of the Discourse-Voice Regulatory Configurations

##### Cluster 1: the “general therapeutic work” discourse-voice regulatory configuration

**FIGURE 3:**
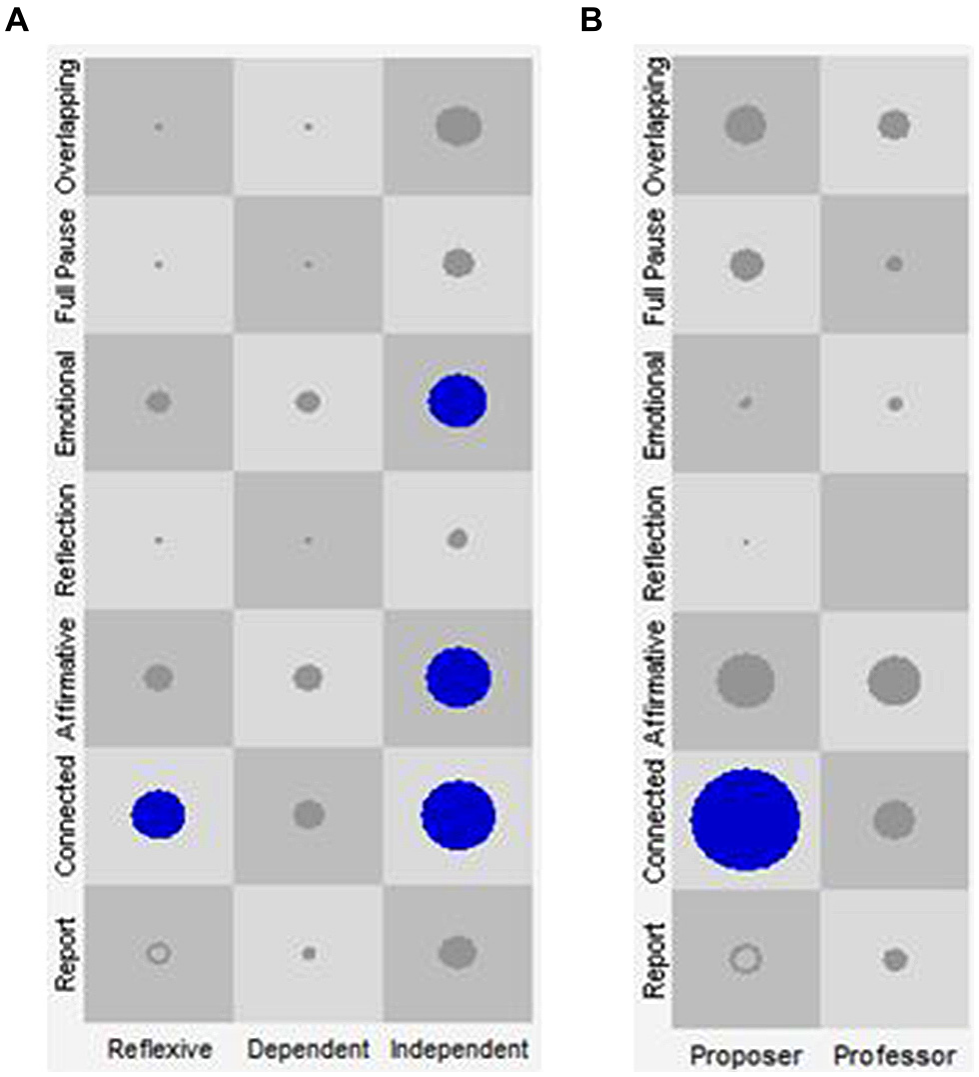
**SSG of Cluster 1: general therapeutic work.** The *X*-axis represents the discursive positions of the patient (**A**: Reflexive, Dependent and Independent) and of the therapist (**B**: Proposer and Professor). The *Y*-axis represents the VQPs for patient and therapist. Each one of the cells represents all the state space resulting from the combination of the discursive positions with the VQPs. The circles and their size represent the visits in each cell and their frequency, respectively. Only the cells in blue represent the identified attractors.

This configuration seems to indicate an exploratory and deconstructive therapeutic activity in which different discursive positions participate, shaped by a vocal combination that conveys the impression of connection with the other, and at the same time, a strong conviction and elaboration of what is being said. Specifically, in the case of the patient, the Independent discursive position co-occurred with a wide range of regulatory resources in terms of vocal quality (Connected, Affirmative, and Emotional VQPs). This gives the impression that this position—one that signals that the patient probably needs therapeutic help—is in tension between elaboration, emotional regulation, and conviction. The Dependent discursive position and its prosodic characteristics, however, do not occur at all in the General Therapeutic Work configuration. Finally, the Reflexive discursive position appears together with the Connected VQP as a prosodic characteristic that displays connection with the other and an orientation toward elaboration. In the case of the therapist, in this configuration the Proposer discursive position appears in combination with the Connected VQP, a vocal quality that conveys elaboration and an orientation toward the interlocutor (see **Table 6** and **Figure 3**).

**Table 6:** Total visits for each discursive position- VQP combination in patients and therapists of each cluster.

	Discursive positions/VQPs	Report	Connected	Affirmative	Reflexive	Emotional	Full pause	Overlapping	Total
Cluster 1	**Patient**
	Reflexive	7 (1.62%)	46^∗^ (10.69%)	16 (3.72%)	2 (0.46%)	10 (2.32%)	2 (0.46%)	2 (0.46%)	
	Dependent	4 (0.93%)	18 (4.18%)	14 (3.25%)	2 (0.46%)	10 (2.32%)	2 (0.46%)	2 (0.46%)	
	Independent	24 (5.58%)	89^∗^ (20.69%)	67^∗^ (15.58%)	8 (1.86%)	54^∗^ (12.55%)	18 (4.18%)	33 (7.67%)	430
	**Therapist**
	Proposer	10 (3.10%)	141^∗^ (43.78%)	41 (12.73%)	1 (0.31%)	2 (0.62%)	16 (4.96%)	22 (6.83%)	
	Professor	9 (2.79%)	23 (7.14%)	36 (11.18%)	0 –	3 (0.93%)	4 (1.24%)	14 (4.34%)	322
Cluster 2	**Patient**
	Reflexive	8 (10.38%)	1 (1.29%)	0 –	0 –	1 (1.29%)	1 (1.29%)	0 –	
	Dependent	42^∗^ (54.54%)	10 (12.98%)	1 (1.29%)	3 (3.89%)	6 (7.79%)	2 (2.59%)	0 –	
	Independent	1 (1.29%)	0 –	0 –	0 –	0 –	0 –	1 (1.29%)	77
	**Therapist**
	Proposer	10^∗^ (16.39%)	7^∗^ (11.47%)	0 –	1 (1.63%)	0 –	9^∗^ (14.75%)	0 –	
	Professor	14^∗^ (22.95%)	9^∗^ (14.75%)	1 (1.63%)	0 –	1 (1.63%)	9^∗^ (14.75%)	0 –	61
Cluster 3	**Patient**
	Reflexive	13 (2.69%)	131^∗^ (27.12%)	69^∗^ (14.28%)	19 (3.93%)	22 (4.55%)	38 (7.86%)	17 (3.51%)	
	Dependent	2 (0.41%)	19 (3.93%)	14 (2.89%)	3 (0.62%)	8 (1.65%)	1 (0.20%)	4 (0.82%)	
	Independent	9 (1.86%)	35 (7.24%)	33 (6.83%)	6 (1.24%)	16 (3.31%)	13 (2.69%)	11 (2.27%)	483
	**Therapist**
	Proposer	4 (0.82%)	100^∗^ (20.53%)	115^∗^ (23.61%)	4 (0.82%)	5 (1.02%)	59^∗^ (12.11%)	21 (4.31%)	
	Professor	2 (0.41%)	50^∗^ (10.26%)	90^∗^ (18.48%)	2 (0.41%)	3 (0.61%)	20 (4.10%)	12 (2.46%)	487
Cluster 4	**Patient**
	Reflexive	18 (5.02%)	18 (5.02%)	2 (0.55%)	6 (1.65%)	17 (4.74%)	6 (1.65%)	1 (0.27%)	
	Dependent	45^∗^ (12.56%)	48^∗^ (13.40%)	22 (6.14%)	11 (3.07%)	98^∗^ (27.37%)	20 (5.58%)	7 (1.95%)	
	Independent	16 (4.46%)	7 (1.95%)	3 (0.83%)	1 (0.27%)	10 (2.79%)	2 (0.55%)	0 –	358
	**Therapist**
	Proposer	29 (8.35%)	91^∗^ (26.22%)	13 (3.74%)	6 (1.72%)	3 (0.86%)	48^∗^ (13.83%)	5 (1.44%)	
	Professor	21 (6.05%)	71^∗^ (20.46%)	20 (5.76%)	5 (1.44%)	8 (2.30%)	24 (6.91%)	3 (0.86%)	347

The asterisks indicate the identified attractors. Cluster 1, *N* = 44 episodes; Cluster 2, *N* = 11 episodes; Cluster 3, *N* = 56 episodes; Cluster 4, *N* = 40 episodes.

##### Cluster 2: the “disconnected” discourse-voice regulatory configuration

**FIGURE 4:**
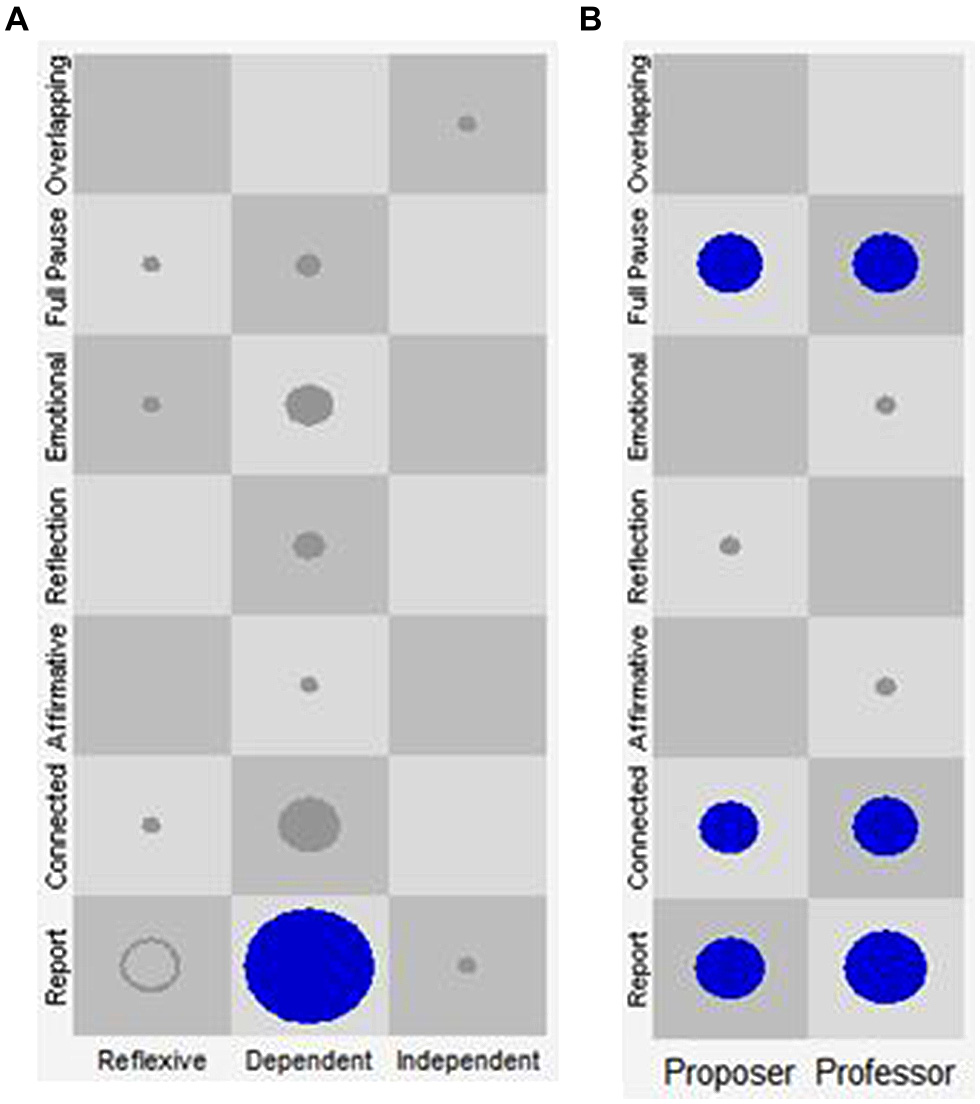
**State space grid (SSG) of Cluster 2: disconnected.** The *X*-axis represents the discursive positions of the patient (**A**: Reflexive, Dependent, and Independent) and of the therapist (**B**: Proposer and Professor). The *Y*-axis represents the VQPs for patient and therapist. Each one of the cells represents all the state space resulting from the combination of the discursive positions with the VQPs. The circles and their size represent the visits in each cell and their frequency, respectively. Only the circles in blue represent the identified attractors.

This configuration appears only in Rupture Episodes of therapy 1 and makes reference to the prevalence of discourse-voice regulatory strategies working as attractors in both participants that conjointly indicate a disconnection of the therapeutic activity (see **Table 6** and **Figure 4**). Five attractors take place in this configuration (one of the patient and six of the therapist), mainly occurring with the Report VQP, giving the impression of a lack of affective commitment with what is being said.

Specifically for the patient, the Dependent discursive position mainly employs the Report VQP. For the therapist, both the Proposer and the Professor discursive positions use mostly the Report and Connected VQPs and the Full Pause category. Particularly, the use of Full Pause in the disconnected configuration—a category that by itself constitutes a regulatory strategy with the interlocutor—seems to account merely for the promotion of continuity in the other’s communication.

##### Cluster 3: the “productive therapeutic work” discourse-voice regulatory configuration

**FIGURE 5:**
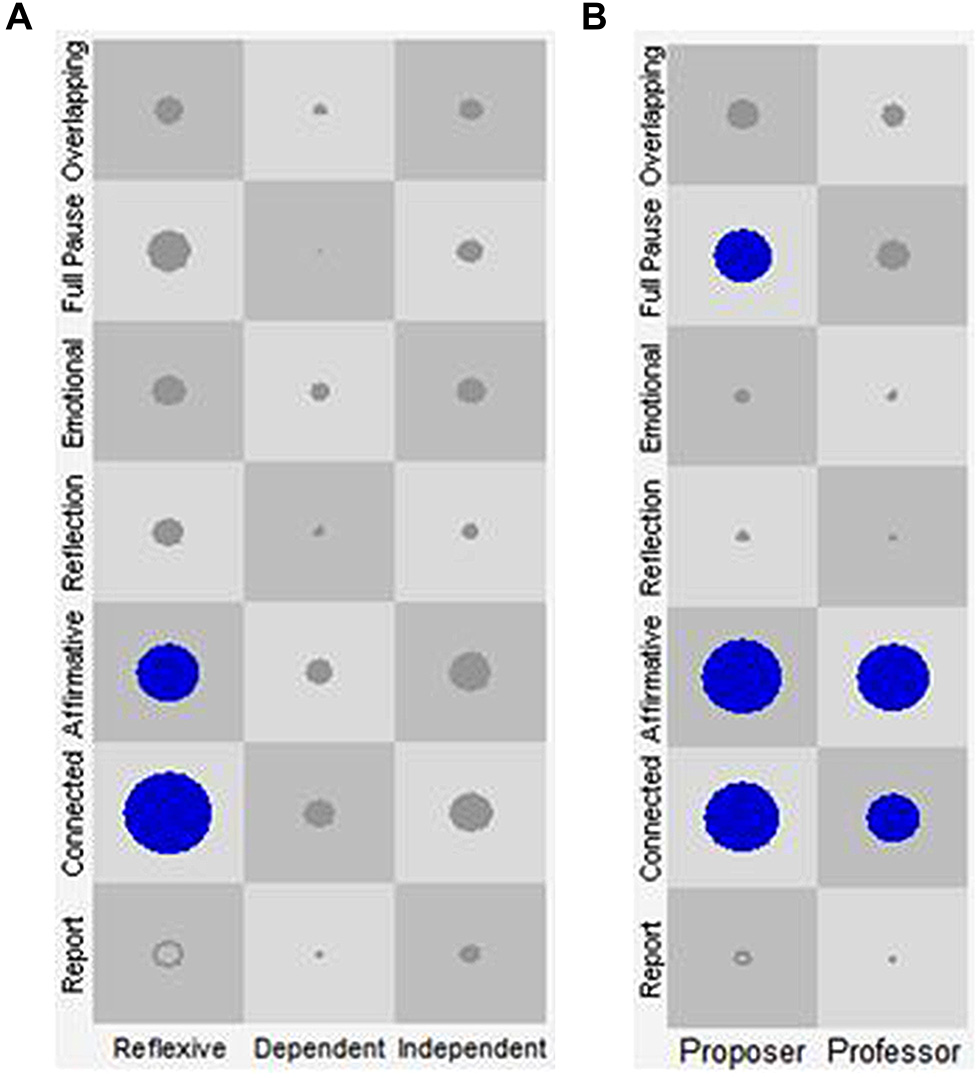
**State space grid of Cluster 3: productive therapeutic work.** The *X*-axis represents the discursive positions of the patient (**A**: Reflexive, Dependent, and Independent) and of the therapist (**B**: Proposer and Professor). The *Y*-axis represents the VQPs for patient and therapist. Each one of the cells represents all the state space resulting from the combination of the discursive positions with the VQPs. The circles and their size represent the visits in each cell and their frequency, respectively. Only the circles in blue represent the identified attractors.

This configuration seems to indicate a productive and constructive therapeutic activity in which several discursive positions participate combined with a prosody that gives the impression of connection with the other, and at the same time, of strong conviction in and elaboration of what is being said (see **Table 6**; **Figure 5**).

Specifically for the patient, the frequent use of the Reflexive discursive position combined with the Connected and Affirmative VQPs as regulatory strategies shows that, in the “Productive Therapeutic Work” regulatory configuration, the Reflexive position is central for the constructive nature of therapeutic work. Regarding the therapist, both the Proposer and the Professor discursive positions are combined with the Affirmative and Connected VQPs. Therefore, overall and in terms of their vocal quality, the therapist’s positions appear to be directed to enhancing the elaborative and constructive characteristics of the regulatory strategies employed by the patient.

##### Cluster 4: the “emotional therapeutic work” discourse-voice regulatory configuration

**FIGURE 6:**
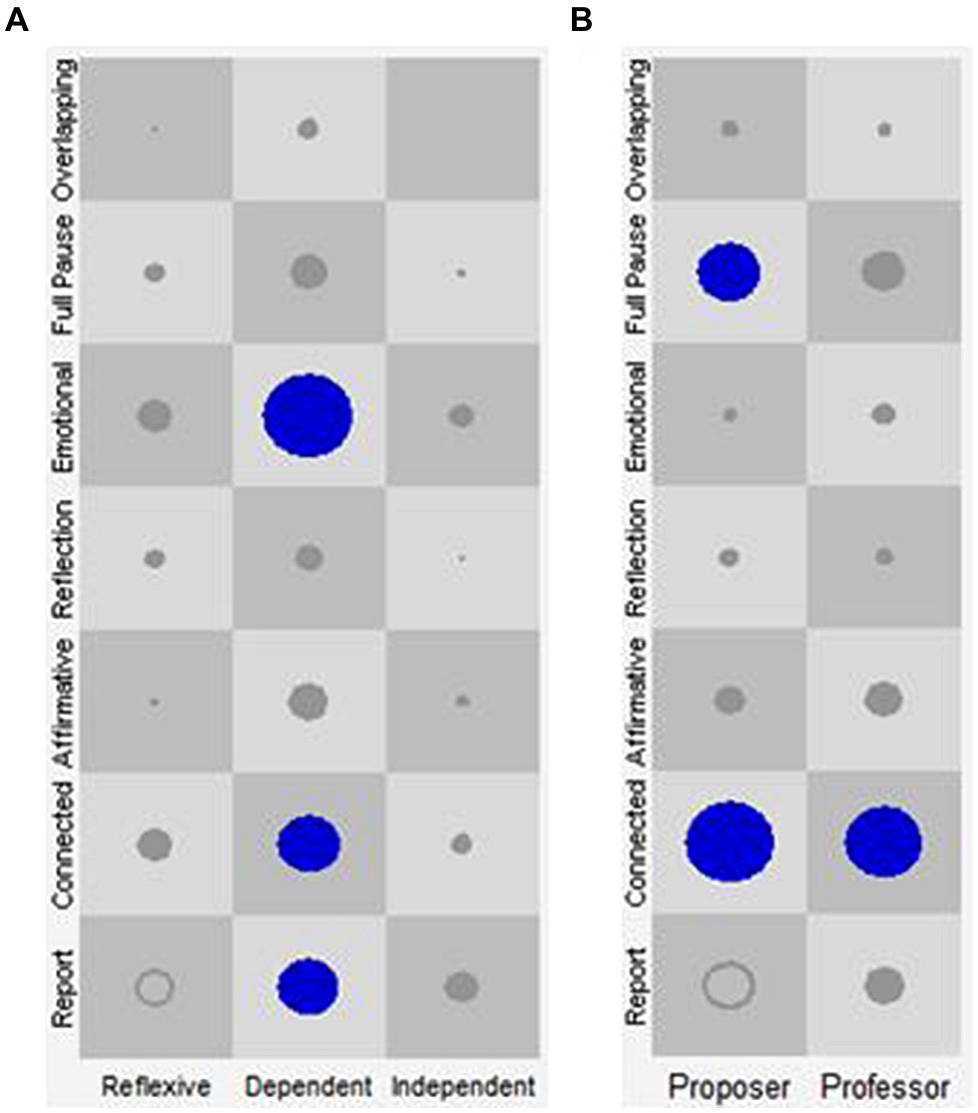
**State space grid of Cluster 4: emotional therapeutic work.** The *X*-axis represents the discursive positions of the patient (**A**: Reflexive, Dependent, and Independent) and of the therapist (**B**: Proposer and Professor). The *Y*-axis represents the VQPs for patient and therapist. Each one of the cells represents all the state space resulting from the combination of the discursive positions with the VQPs. The circles and their size represent the visits in each cell and their frequency, respectively. Only the circles in blue represent the identified attractors.

This configuration refers to the prevalence of discourse-voice regulatory strategies working as attractors that together seem to indicate a therapeutic activity characterized by an affective component. In this configuration, the Dependent discursive position of the patient gives the impression of vocal expression and suppression of emotions in speech, but also of detachment in relation to what is being said (see **Table 6**; **Figure 6**). It seems that the strong presence of the emotional vocal quality is shaped by a more elaborative quality of the speaker’s words and his/her disengagement in the Reflexive and Independent positions, expressed by the Connected and Report VQPs respectively. For the therapist, both the Proposer and the Professor discursive positions are combined with the Connected VQP. This prosodic characteristic could have the function of regulating the affection–disaffection polarity in the patient.

### Configurations of Discourse-Voice Regulatory Strategies: Patterns of Attractors in Relevant Episodes

To test hypothesis 4, the probability of encountering each of the four configurations of discourse-voice regulatory strategies working as attractors (clusters) was compared according to the type of episode. Thus, four dichotomous variables were created for this analysis, acquiring value 1 when an episode (or trajectory) displayed a certain configuration of discourse-voice regulatory strategies (i.e., Cluster 1) and 0 if any of the other configurations were present (e.g., Clusters 2, 3, or 4).

A Logistic Hierarchical Regression analysis (using HLM version 7.0, Full PQL estimation method, Bernoulli distribution at Level-1) in a two-level model was used. In the model, the episodes (Level-1) were nested in the patient (Level-2). The type of Episode was the predictor at Level-1 (0 = Rupture Episode and 1 = Change Episode). The Level 2 predictors were Initial Patient Functionality (Functional; indicating the level of functioning, of the patient at the beginning of the therapy; 0 = Beginning the psychotherapy in the dysfunctional population and 1 = Beginning the psychotherapy in the functional population) and Reliable Change Index in the Patient (RCI, indicating the outcome of the therapy; 0 = without RCI and 1 = with RCI).

Separate HLM analyses were conducted for three of the clusters. Given that cluster 2 appears only in Rupture Episodes of therapy 1, it was not analyzed. Three steps were followed for each analysis:

(a) A fully unconditional model was fitted in order to estimate dependent variable reliability and the adequacy of the multilevel analysis.
(b) The Type of Episode was included in the Level-1 equation and was modeled as a random effect in order to determine if the coefficients varied among patients. If there was no variability to explain, its variance was fixed at zero.
(c) Finally, Initial Patient Functionality and/or Reliable Change Index in the Patient were included as predictors at Level-2 intercept and/or slope (Type of Episode). Whenever these predictors did not explain significant variances of the Level-2 equations, they were also dropped out of the model. **Table 7** presents the final models of each discourse-voice regulatory configuration at the episode level.

**Table 7:** Configurations of discourse-voice regulatory strategies according to type of episode.

Fixed Effects^a^	Cluster 1^c^	Cluster 3^c^	Cluster 4^c^
	Coefficient (SE) Odds (95% CI)	Coefficient (SE) Odds (95% CI)	Coefficient (SE) Odds (95% CI)
Intercept (*γ*_00_)	-0.74 (0.63) 0.48 (0.08–2.78)	-1.20 (0.60) 0.30 (0.06–1.59)	-1.73 (0.33)^∗^ 0.18 (0.06–0.58)
Initial patient functioning (*γ*_01_)^b^	–	–	-2.42 (0.43)^∗^ 0.09 (0.02–0.35)
Type of episode (*γ*_10_)	-0.90 (0.41)^∗^ 0.23 (0.17–0.71)	1.29 (0.44)^∗∗^ 3.62 (1.51–8.70)	0.49 (0.43) 1.64 (0.70–3.87)
**Random variance components**			
Level-2 Intercept(*u*_0_)	1.29^∗∗∗^ 38.01 (4)	1.27^∗∗∗^ 41.11 (4)	0.001^∗^ 8.39 (3)

Level-1 *N*= 151, Level-2 *N*= 5. Type of episode, 0 = Rupture Episodes and 1 = Change Episodes. Functional, 1 = Beginning the psychotherapy in the functional population and 0 = Beginning in the dysfunctional population. ^a^Gamma (γ) coefficients in fixed effects and variance components in random effects. Standard errors (SE) follow parameter estimates in parentheses and 95% Confidence Interval (CI) Odds Ratio follow Odds Ratio estimator in parentheses (Fixed Effects). Ch^2^ and *df* below variances in parentheses (Random effects). ^b^Initial Patient Functioning centered around the grand mean. Final Models: ^c^Log (Probability of Dependent Variable) = γ_00_ + γ_10_^∗^Type of Episode+ u_0_; Log (Probability of Dependent Variable) = γ_00_ + γ_01_^∗^Functional + γ_10_^∗^Type of Episode + u_0_. ^∗^*p* < 0.05, ^∗∗^*p* < 0.01, ^∗∗∗^*p* < 0.001.

The results indicate that the “Productive Therapeutic Work” configuration (cluster 3) showed statistically significant differences in its probability of occurrence according to the type of episode. Specifically, this configuration was more likely to appear in Change Episodes (Odds ratio 3.62, 95% CI 1.51; 8.70). The opposite happens in the case of the “General Therapeutic Work” configuration (cluster 1), which was used more frequently in Rupture Episodes (Odds ratio 0.23, 95% CI 0.17; 0.71). Meanwhile, the probability of occurrence of the “Emotional Therapeutic Work” configuration (cluster 4) did not show significant differences regardless of the type of episode considered.

## Discussion

The questions addressed in this study were whether it was possible to observe discourse-voice regulatory strategies working as attractors—along with configurations of these strategies—and also if these configurations of recurrent and stable discourse-voice regulatory strategies changed in different interactional scenarios and over the psychotherapeutic process.

In the case of this study, we observed the dynamics of verbal and non-verbal behaviors in the psychotherapeutic interaction by means of a pattern analysis (Salvatore and Tschacher, 2012). Specifically, using the SSG, we analyzed the co-occurrence of discursive and vocal behaviors considering the relationship among them. By means of this method, we explored all possible states of the analyzed system (i.e., combinations of each Discursive Position with all VQPs). The chosen organization level was one that combined micro-elements (i.e., VQPs) nested within a more macro organization (i.e., discursive positions). This system was comprised of moment-by-moment dynamics (i.e., the patient–therapist interaction) and was nested within a larger social structure (i.e., the psychotherapy; Hollenstein, 2007). Thus, the method of analysis used in this study, inspired by the DST, allowed the results to acquire ecological validity and clinical value when considering and modeling them as phenomena that emerge from the dynamics of the elements of a complex system: the psychotherapy.

The results show that the regulation occurs between the participants of the therapeutic dyad, and also between different dimensions of the behavior of each one of them (i.e., verbal and non-verbal). In this regard, discourse-voice regulatory strategies are considered to be discursive expressions of the multiple subjectivity in patient–therapist interaction which are modulated non-verbally by vocal qualities.

With respect to our first hypothesis, the recurrent and stable discourse-voice regulatory strategies observed matched theoretical definitions in some cases (Triadic Model of Positioning- Martínez et al., 2014b; VOQs- Tomicic et al., 2011) and empirical results in others (e.g., Martínez et al., 2014c; Tomicic et al., 2014). However, the hypothesized combination Independent position-Affirmative VQP was not confirmed. Also, four unexpected discourse-voice regulatory strategies working as attractors were observed: three for the patients (Reflexive position-Affirmative VQP as well as Dependent position-Report and Connected VQPs), and one for the therapists (the Professor position-Connected VQP). These combinations may be self-regulatory phenomena associated with specific moments of the psychotherapeutic interaction and not necessarily predictable from the theory.

On the other hand, the Dependent and Independent discursive positions—that is, those aspects of the patient’s self related to his/her psychological problems that motivated him/her to seek psychotherapeutic help—present a less consistent non-verbal regulatory profile, at least with respect to their prosodic characteristics (e.g., no attractors were found in the case of the Independent discursive position of the patients). Also, this could be associated with the typical variability of patients’ non-verbal expressions as observed by Tomicic et al. (2014).

Related with the second hypothesis, the results show that discourse-voice regulatory strategies working as attractors change as the interactional scenario changes. This can be understood considering that the dynamics (time, self-organization) associated with the regulatory processes represent the emergence of recurring patterns of association of different patient–therapist behaviors within interactional scenarios. Therefore, with regard to the use of the regulatory strategies in Change Episodes and Rupture Episodes, the consistent association established between the specific verbal and non-verbal expressions of the regulation within these different interactional scenarios helps to comprehend the dynamic and emergent nature of the psychotherapeutic interaction. This was differently observed in several attractors (i.e., recurrent and stable discourse-voice regulatory strategies) depending on the type of episode in which they were analyzed. The fact that the combination of the Reflexive discursive position of the patient with the Connected and Affirmative VQPs was more prevalent in Change Episodes indicates that the patient’s prosody—elaborative and oriented toward the other, and simultaneously, with a quality of certainty and conviction—is coherent with the dialogical characteristics of this discursive position and also with the construction of a new subjective theory, a notion underlying Change Episodes.

In terms of dynamic systems, as was mentioned before, these interactional scenarios—Change Episodes and Rupture Episodes—are interpreted as part of a bigger system (all the sessions of a psychotherapeutic process) which shows phase transitions, that is to say, transformations at a structural level or reconfigurations of the state-space (Hollenstein, 2007). In this study, these phase transitions were characterized by reorganizations of the elements when the system was strained, for example the emergence of some specific discourse-voice regulatory strategies working as attractors in a Rupture Episode. Then, in the reorganization process, the system could return to the previous organization or present a new one. This was true for Change Episodes, in which a variety of these attractors were observed. In the case of the therapists, the same can be said for the regulatory strategy Proposer position-Connected VQP, in which the consistency between its discursive and prosodic dimensions reveals a specific therapeutic activity deployed in this type of interactional scenarios.

On the other hand, confirming the third hypothesis, different configurations of recurrent and stable discourse-voice regulatory strategies were identified. These could be interpreted as a self-organized group of discourse-voice regulatory strategies working as attractors that constitutes an interactional system with its own characteristic global property (i.e., configuration as a whole; Fogel, 2006, 2011). From this perspective, the same discursive position combined with the same vocal quality would acquire distinct meanings and regulatory functions in two different configurations of regulatory strategies. And, in this respect, as in the case of relevant episodes, these configurations would themselves constitute an interactional scenario with their own purposes and results. This global property allows us to comprehensibly describe the dynamic behaviors of the configurations of discourse-voice regulatory strategies of the therapeutic relationship as a complex system that involves different levels of organization, from the specific psychotherapeutic moment to the cultural conditions in which this system is embedded (Salvatore and Tschacher, 2012; Martínez et al., 2014a).

Finally, we can confirm the fourth hypothesis, in which we expected to find that different configurations of patients’ and therapists’ discourse-voice regulatory strategies working as attractors would not be observed in the same proportion in Change Episodes and Rupture Episodes. Specifically, we found that the “Productive Therapeutic Work” configuration was more probable in Change Episodes, whereas the “General Therapeutic Work” configuration was more probable in Rupture Episodes. On the one hand, it was fairly expectable that the “Productive Therapeutic Work” configuration would emerge more frequently in Change Episodes, since in this type of interactional scenario participants have been observed to deploy behaviors that tend to show attunement, dialogicity, and collaborative work toward the therapeutic aims (Arístegui et al., 2009; Dagnino et al., 2012; Fernández et al., 2012; Tomicic et al., 2014). On the other hand, in a difficult and tense interactional scenario such as a Rupture Episode, it makes sense for the patient to display several regulatory strategies. But what is remarkable is that the therapist uses the other configuration in the same way, acquiring in this case a different regulatory function. As Safran and Kraus (2014) point out, specific interpersonal behaviors and subtle non-verbal cues on the part of the patient can “tug” or “pull” the therapist in a rupture which particularly taxes his/her therapeutic role.

With respect to the limitations of this study, we must highlight the small size of the sample (five patients) and the heterogeneity in the length of the therapies (short and long term psychotherapies were analyzed together). This shortcoming, which made it impossible to model the patient level, could not be solved by considering the functionality of the participants before and after the therapy. On the other hand, regarding the different lengths of the therapies, one could ask if the fact that no association was found between the configurations of recurrent and stable discourse-voice regulatory strategies and their deployment over time has anything to do with the different dynamics which are reasonable to expect in short term therapeutic process versus long term ones. For this reason, these findings must be carefully interpreted when weighing the possibility of generalizing themed *vis-a-vis*. However, considering that these configurations can emerge from the particular characteristics of a therapeutic dyad (e.g., the case of the disconnected configuration), it is relevant to describe them to understand the various forms that verbal and non-verbal regulation can acquire, and, in turn, to specify the meaning of such regulations for these interactions and therapeutic interventions. In this regard, it is possible to hypothesize that the levels of complexity in the observations increase from regulatory strategies to configurations of groups of these strategies. That is to say, the distinctions became more specific and idiographic in scope. Despite this, the results regarding configurations of discourse-voice regulatory strategies working as attractors do not appear to be random, and in terms of their regulatory functions, are also consistent with the settings where they are deployed.

The microscopic observations carried out in this study account for implicit interaction processes that are not necessarily part of the conscious experience of the therapists in their clinical practice. However, the results presented here could be useful for them to make distinctions regarding the regulatory functions of different combinations of discursive and prosodic features in the interaction with their patients. This information may allow psychotherapists, for example, to extend their therapeutic listening and assess the state of the relationship with their patients and its variations throughout the psychotherapeutic process.

## Conflict of Interest Statement

The authors declare that the research was conducted in the absence of any commercial or financial relationships that could be construed as a potential conflict of interest.

## Footnotes

1 Here, we are using the term “strategy” in its original meaning as a noun: “A method that is worked out in advance for achieving some objective [in this case self or mutual regulation aim]” (Merriam-Webster). Since it is not a verb, the strategy constitutes not an action of the individual, but an object or a tool that the individuals use, consciously or not consciously, to achieve a goal.

2 The Avoidant discursive position was not analyzed in detail because it was observed in only two of the analyzed psychotherapies.

3 Because episodes are nested in the sessions (that is, a session may have one or more events), we explored whether there was variability associated with this level. We found that none of the dependent variables considered showed variability in association with the session level. The information on the upper level (patient) was collapsed.

4 Given the small sample size of patients (*N* = 5) that could be used to reveal differences in the behavior of the episodes in terms of discourse-voice strategies, the predictors of initial functionality and the RCI were modeled at level 2 as a way to resolve the difficulty of accounting for the impact that the differences among patients might have on the results.

5 Both indicators were measured using the Outcome Questionnaire OQ-45.2 (Lambert and Burlingame, 1996).

6 Two episodes/trajectories were left out of the analysis because they were outliers: one Rupture Episode from session 5 and one Rupture Episode from session 13, both from therapy 4.
